# A novel transcription factor specifically regulates GH11 xylanase genes in *Trichoderma reesei*

**DOI:** 10.1186/s13068-017-0878-x

**Published:** 2017-08-03

**Authors:** Rui Liu, Ling Chen, Yanping Jiang, Gen Zou, Zhihua Zhou

**Affiliations:** 1 CAS-Key Laboratory of Synthetic Biology, CAS Center for Excellence in Molecular Plant Sciences, Institute of Plant Physiology and Ecology, Chinese Academy of Science, Fenglin Rd 300, Shanghai, 200032 China; 2 University of Chinese Academy of Sciences, Beijing, 100049 China

**Keywords:** Hemi-cellulase, Transcription factor, Xylanase, Glycoside hydrolase, *Trichoderma reesei*, CRISPR/Cas9

## Abstract

**Background:**

The filamentous fungus *Trichoderma reesei* is widely utilized in industry for cellulase production, but its xylanase activity must be improved to enhance the accessibility of lignocellulose to cellulases. Several transcription factors play important roles in this progress; however, nearly all the reported transcription factors typically target both cellulase and hemi-cellulase genes. Specific xylanase transcription factor would be useful to regulate xylanase activity directly.

**Results:**

In this study, a novel zinc binuclear cluster transcription factor (jgi|Trire2|123881) was found to repress xylanase activity, but not cellulase activity, and was designated as SxlR (specialized xylanase regulator). Further investigations using real-time PCR and an electrophoretic mobility shift assay demonstrated that SxlR might bind the promoters of GH11 xylanase genes (*xyn1*, *xyn2*, and *xyn5*), but not those of GH10 (*xyn3*) and GH30 (*xyn4*) xylanase genes, and thus regulate their transcription and expression directly. We also identified the binding consensus sequence of SxlR as 5′- CATCSGSWCWMSA-3′. The deletion of SxlR in *T. reesei* RUT-C30 to generate the mutant ∆*sxlr* strain resulted in higher xylanase activity as well as higher hydrolytic efficiency on pretreated rice straw.

**Conclusions:**

Our study characterizes a novel specific transcriptional repressor of GH11 xylanase genes, which adds to our understanding of the regulatory system for the synthesis and secretion of cellulase and hemi-cellulase in *T. reesei*. The deletion of SxlR may also help to improve the hydrolytic efficiency of *T. reesei* for lignocellulose degradation by increasing the xylanase-to-cellulase ratio.

**Electronic supplementary material:**

The online version of this article (doi:10.1186/s13068-017-0878-x) contains supplementary material, which is available to authorized users.

## Background

Lignocellulosic biomass, consisting mostly of cellulose, hemi-cellulose, and lignin, is the most abundant and renewable energy source on earth [1]. Degradation of lignocellulosic biomass and continuation of the carbon cycle in nature is maintained mainly by microbial action, including different fungal species, such as *Trichoderma, Aspergillus*, *and Penicillium*. The biomass-degrading enzymes produced by these organisms also have applications in various fields of industry including food, fodder, paper, and textile industries. *Trichoderma reesei* is a well-known efficient producer of cellulase and hemi-cellulase, and is therefore widely employed by the enzyme industry for production of its own endogenous enzymes as well as production of heterogeneous proteins [2].

Cellulosic biofuel production continues to increase world-wide every year with a consequent rapidly increasing requirement for cellulase production from *T. reesei*. However, the production and optimization of enzyme formulations for lignocellulose degradation are still the major barriers to its extensive application. It is necessary to further enhance the hydrolytic efficiency of *T. reesei* enzyme preparations for lignocellulose and lower their relatively high cost. Due to the complex constitution and structure of native lignocellulose, the enzyme preparations produced by *T. reesei* must be supplemented with several types of exogenous enzymes to achieve effective degradation of natural complex lignocellulosic materials. For example, exogenous hemi-cellulase and other auxiliary enzymes are added to commercial cellulase complexes from Danisco or Novozymes [3]. This indicates that increasing the production of the most prominent hemi-cellulase (endo-β-1,4-xylanase), which catalyzes the hydrolysis of 1,4-β-d-xylosidic linkages in xylan to short xylooligosaccharides of varying length in *T. reesei*, is a good way to strengthen its hydrolysis activity [4].

Expression of genes encoding cellulase and hemi-cellulase in *T. reesei* is tightly controlled at the transcriptional level. Therefore, deleting and/or over-expressing transcription factors (TFs) that specifically regulate xylanase gene expression in *T. reesei* is a straightforward way to perform knowledge-based strain design. However, most known TFs regulate expression of both cellulase and xylanase genes in the same way. The most extensively studied TF is the negative TF CRE1 [5], which mediates carbon catabolite repression (CCR). In the cellulase hyperproduction strain *T. reesei* RUT-C30, CRE1 is truncated, which renders this strain carbon catabolite depressed [6]. The global transcriptional activator Xyr1 is obligatory for expression of most cellulase and hemi-cellulase genes [7, 8]. Other recognized TFs are the positively acting factor ACE2 [9, 10] and HAP2/3/5 complex [11], and the negatively acting factor ACE1 [12]. In addition, BglR [13] was identified as a new TF that upregulates expression of specific genes encoding β-glucosidases in *T. reesei*, and a putative methyltransferase, LAE1 [14, 15], was found to be essential for cellulase and hemi-cellulase production in *T. reesei*, although the mechanism is still unclear. ACE3 has been newly characterized to be indispensable for cellulase and xylanase activity in *T. reesei* [16]. All the above TFs have effects on the expression of both cellulase and hemi-cellulase genes.

Recently, a study found that some segmentally aneuploid (SAN) *T. reesei* strains exhibited enhanced growth in xylan-based media and produced higher levels of xylanase [17]. Further analysis confirmed that D segment duplication, not L segment deletion, in the genomes of these SAN strains, was responsible for the growth advantage in xylan-based media, but did not affect the cellulase activity. In fact, the Xpp1, TF, was identified by a pull-down assay based on the *xyn2* promoter and was confirmed as a negative regulator of the *xyn1* and *xyn2* xylanase genes and the *bxl2* β-xylosidase gene [18].

In this study, using bioinformatics analysis and gene deletion with the CRISPR/Cas9 system [19], we identified a gene encoding a protein designated as specialized xylanase regulator (SxlR). Deletion of the *sxlr* gene resulted in increased xylanase activity while not affecting cellulase activity. This indicated that SxlR might be a novel TF that regulates xylanase expression. According to the results of real-time PCR (qPCR) and electrophoretic mobility shift assay (EMSA) analyses, we demonstrated that SxlR plays a critical role in the inhibition of the expression of xylanases belonging to the glycoside hydrolase 11 family (GH11) in *T. reesei* through binding to the promoter regions of target genes directly. Finally, we identified the binding consensus sequence of SxlR as 5′-CATCSGSWCWMSA-3′.

## Results

### Screening the putative xylanase-specific TFs

**Fig. 1:**
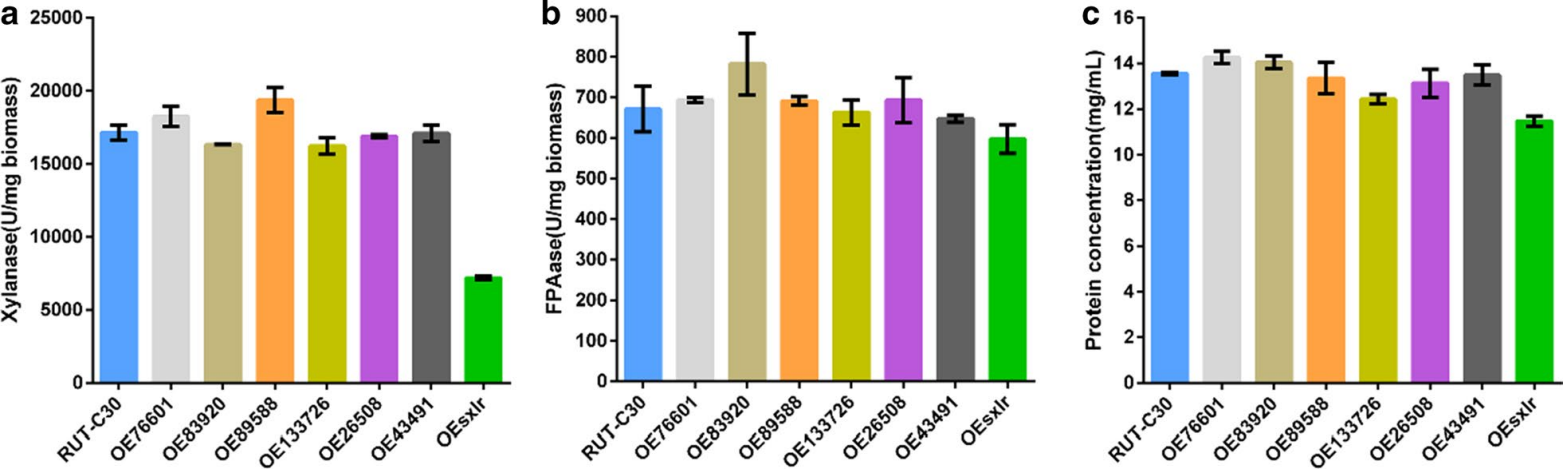
Enzyme activities and proteins of supernatants from *T. reesei* RUT-C30 recombinant strains overexpressing candidate transcription factors. Xylanase activity (**a**), cellulase activity (**b**), and extracellular protein concentration (**c**) of 7-day culture supernatant with wheat bran and Avicel as carbon source. *Error bars* represent the standard deviation of three biological replicates

Based on bioinformatics analysis, we chose seven putative TFs as candidates (Additional file 1) for screening. All these genes were located in five different chromosomes [20, 21], and six of candidates were located in the D segment duplication resulting in higher xylanase activities as reported by Chen et al. [17]. We overexpressed these genes in *T. reesei* RUT-C30. After creating monoconidial cultures for genetic stability, we measured the xylanase and cellulase activities of the transformants. The xylanase activity, but not the cellulase activity, of the strain overexpressing the *sxlr* gene (jgi|TrireRUTC30_1|26638, O*sxlr*) decreased significantly (*t* test, *P* < 0.05) (Fig. 1a, b), and its extracellular protein concentration was decreased (Fig. 1c), which might indicate that this gene affects the xylanase activity alone. In contrast, no changes in xylanase activity, cellulase activity, or protein concentration were detected between RUT-C30 and the other six overexpression strains (Fig. 1).

**Fig. 2:**
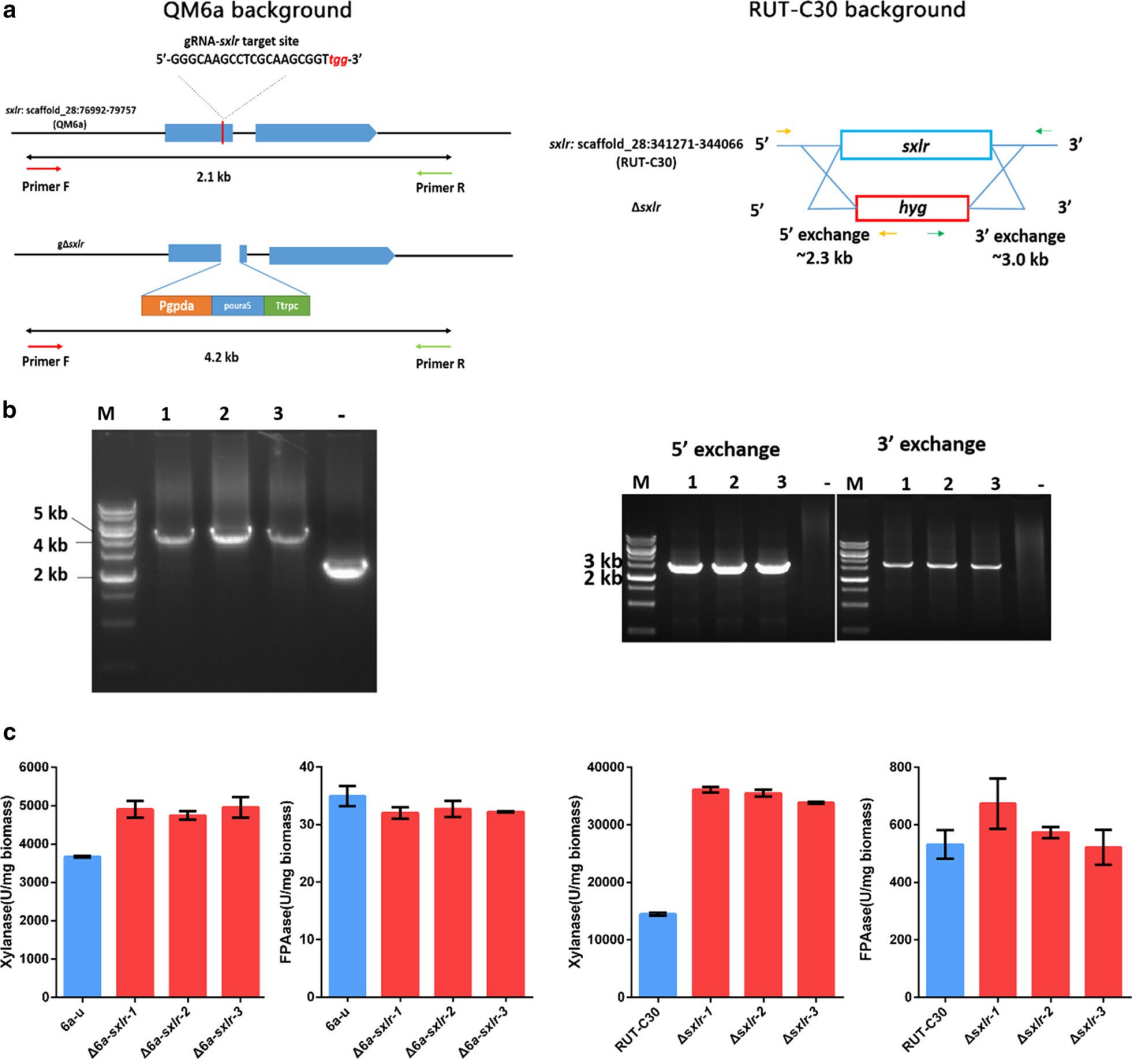
Deletion of *sxlr* in *T. reesei.* **a** Schematic diagram of *sxlr* deletion in *T. reesei* 6a-u and RUT-C30. We used CRISPR/Cas9 system in the *T. reesei* QM6a background and the traditional method in the *T. reesei* RUT-C30 background. **b** PCR results for transformant identification. Three different transformants were chosen for verification. **c** The xylanase and cellulase activity of 7-day culture supernatant with wheat bran and Avicel as carbon sources. *Error bars* represent the standard deviation of three biological replicates

To investigate how the *sxlr* gene is involved in regulation of xylanase activity in *T. reesei*, we deleted *sxlr* gene from the RUT-C30 strain to obtain Δ*sxlr* transformants (Fig. 2a, b). In contrast to the O*sxlr* strain, the xylanase activity of the Δ*sxlr* strains was increased significantly (Fig. 2c). However, the cellulase activity of the Δ*sxlr* strains was almost the same as the parent RUT-C30 strain. We also deleted the homologous *sxlr* gene of *T. reesei* Qm6a (jgi|Trire2|123881, Δ*6a*-*sxlr*) using the CRISPR/Cas9 system to verify the common regulation of xylanase activity in *T. reesei* strains. As expected, the xylanase activity of Δ*6a*-*sxlr* transformants was also dramatically increased, similar to the Δ*sxlr* strain (Fig. 2c), while its cellulase activity was not affected. Taken together, these results suggested that *sxlr* encoded a TF that negatively regulates xylanase activity.

### The deletion of SxlR in *T. reesei* RUT-C30 results in higher xylanase activity and higher reducing sugar yield

**Fig. 3:**
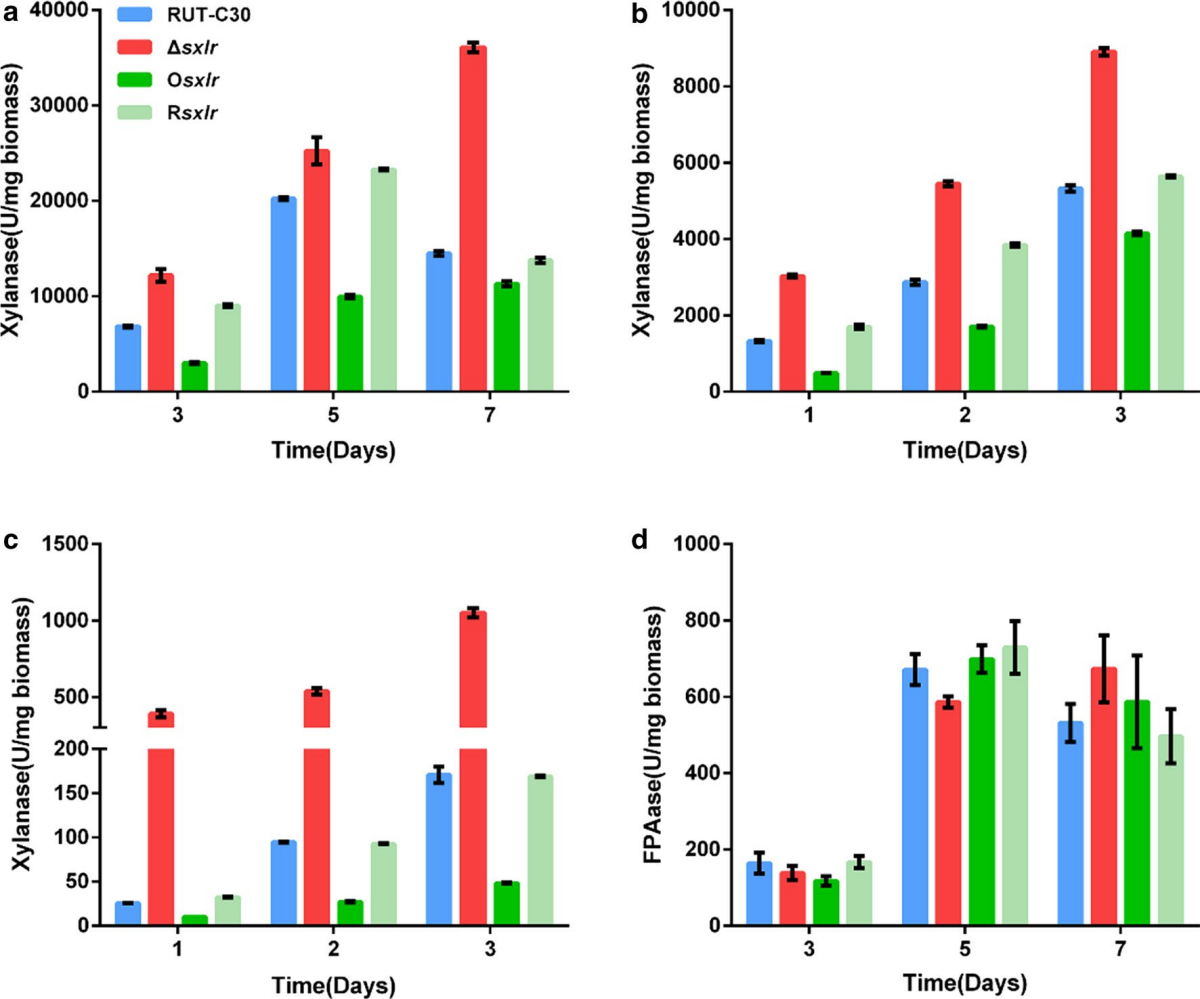
Enzyme activities of *sxlr* transformants on various carbon sources. The xylanase activity of culture supernatants with wheat bran and Avicel (**a**), xylan (**b**), and lactose (**c**) as carbon sources. **d** The cellulase activity of 3-, 5-, and 7-day culture supernatants with wheat bran and Avicel as carbon source. *Error bars* represent the standard deviation of three biological replicates

We selected the cellulase hyperproduction strain RUT-C30 to explore the potential regulatory mechanism of SxlR in *T. reesei.* In addition to the *sxlr* deletion strain (Δ*sxlr,* transformant 1) and the *sxlr* overexpression strain (O*sxlr*), we also constructed the in situ re-complementation strain (R*sxlr*) based on the Δ*sxlr* strain (Additional file 2). The xylanase activity of the Δ*sxlr* strain increased relative to that of RUT-C30 by 0.7-fold and 1.4-fold after 3 and 7 days, respectively, of incubation in inducing medium containing wheat bran and Avicel (Fig. 3a). The xylanase activity was also examined when xylan or lactose was used as the inducer (the sole carbon source in the inducing medium). The xylanase activity of Δ*sxlr* was 1.3-, 0.9-, and 0.7-fold higher than RUT-C30 after 1, 2, and 3 days, respectively, of cultivation with xylan as the inducer (Fig. 3b), and 14.2-, 4.7-, and 5.2-fold higher, respectively, with lactose as the inducer (Fig. 3c). The xylanase activity of the *sxlr* re-complementation control strain R*sxlr* reverted to the same level as that of RUT-C30. In contrast, the *sxlr* overexpression strain O*sxlr* demonstrated weaker xylanase activity than RUT-C30 (Fig. 3a–c). However, no significant difference in cellulase activity was detected between the four strains (Fig. 3d).

**Fig. 4:**
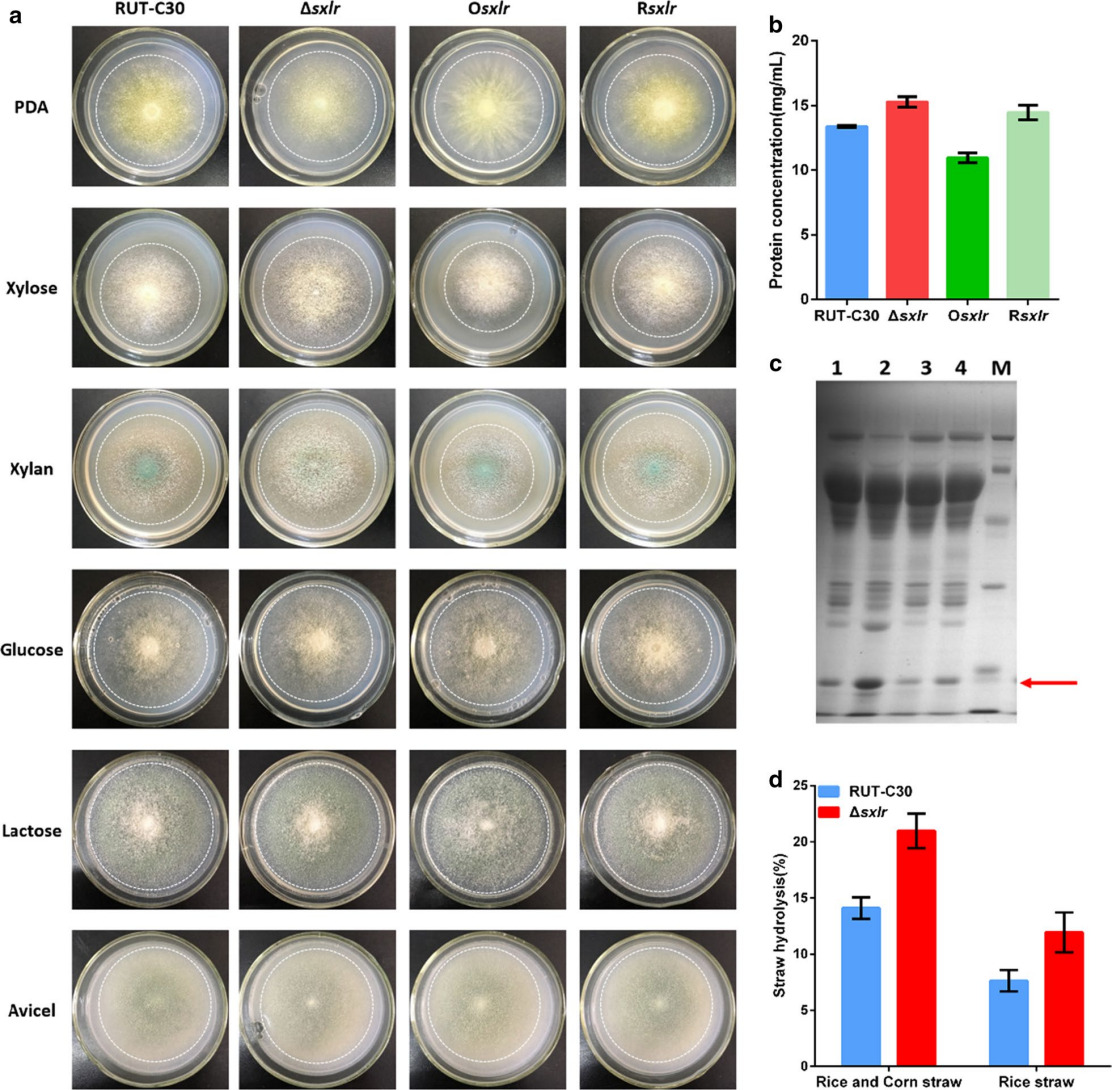
The phenotypes and extracellular protein of *sxlr* transformants. **a** RUT-C30, Δ*sxlr*, O*sxlr*, and R*sxlr* strains grown on PDA plates (3 days) and minimal medium plates containing various carbon sources (1%): xylose (4 days), xylan (4 days), glucose (4 days), lactose (6 days), and Avicel (6 days). Extracellular protein concentration (**b**) and SDS-PAGE analysis (**c**) of supernatants from RUT-C30, Δ*sxlr*, O*sxlr*, and R*sxlr* grown with wheat bran and Avicel as the carbon source. In **c**, *lines 1 to 4* are the supernatants of RUT-C30, Δ*sxlr,* O*sxlr*, and R*sxlr*, respectively. *Line M* is a protein molecular weight marker. **d** The straw hydrolysis reaction was carried out for 3 days, as described in the "Methods" section. *Error bars* represent the standard deviation of three biological replicates

Degradation of lignocellulose is a growth-associated process. The growth rate affects the secretion of enzymes directly [22]. We observed the growth of RUT-C30 and its derivative strains on potato dextrose agar (PDA) and minimal medium (MM) containing 1% xylose, xylan, glucose, lactose, or Avicel as carbon sources, respectively (Fig. 4a). RUT-C30 and its derivative strains did not show significantly different growths and sporulations on PDA and MM containing 1% glucose, lactose or Avicel (Fig. 4a). However, the Δ*sxlr* strain demonstrated rapid growth and the O*sxlr* strain demonstrated reduced growth on MM containing xylose or xylan when compared to RUT-C30. These results could be explained by differences in xylanase activities.

Enzyme activities are also influenced by the concentration and composition of lignocellulose preparations. The extracellular protein concentrations of the Δ*sxlr*, O*sxlr*, and RUT-C30 strains were 15.275 ± 0.096, 10.943 ± 0.399, and 13.378 ± 0.240 mg/mL, respectively. The Δ*sxlr* strain had the highest extracellular protein concentration, and O*sxlr* had the lowest (Fig. 4b). According to sodium dodecyl sulfate–polyacrylamide gel electrophoresis (SDS-PAGE), the band corresponding to ~20 kDa, which was identified as the xylanase protein XYN2 by MALDI-TOF/TOF, was enhanced dramatically in the Δ*sxlr* strain but decreased in the O*sxlr* strain (Fig. 4c). This indicates that SxlR affects the amount of secreted XYN2 in the lignocellulose preparations produced by *T. reesei*.

To determine whether the deletion of SxlR in *T. reesei* RUT-C30 could improve the strain’s efficiency to hydrolyze pretreated lignocellulose, steam-exploded rice straw and steam-exploded rice straw mixed with corn straw were used as saccharification substrates. The crude enzyme complex of the mutant Δ*sxlr* strain produced more reducing sugar than that of RUT-C30 (Fig. 4d). The straw hydrolysis increased to 21% by the supernatant of the Δ*sxlr* strain, while 14.1% by the supernatant of RUT-C30 strain after 3 days of hydrolysis from the pretreated rice and corn straw. Similarly, the straw hydrolysis increased to 11.9% by the supernatant of the Δ*sxlr* strain, while 7.6% by the supernatant of RUT-C30 strain after 3 days of hydrolysis from the pretreated rice straw.

### SxlR regulates the GH11 xylanase genes

**Fig. 5:**
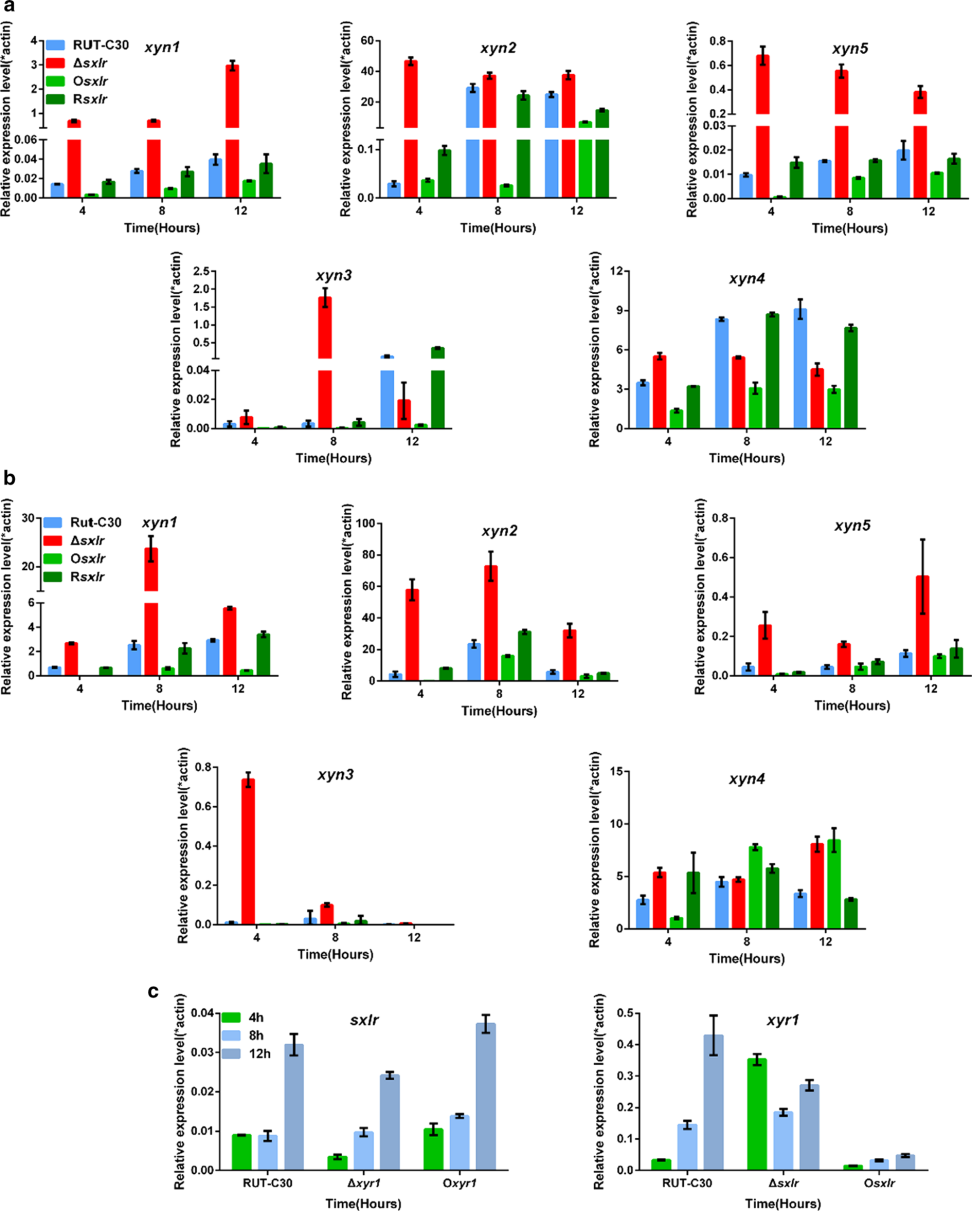
Quantitative PCR analysis of gene expression levels. The expression levels of *xyn1* (GH11), *xyn2* (GH11), *xyn5* (GH11), *xyn3* (GH10), and *xyn4* (GH30) in RUT-C30, Δ*sxlr*, O*sxlr*, and R*sxlr* when wheat bran and Avicel (**a**) and xylan (**b**) were used as carbon sources, expression levels were normalized to the signal of rpl6e. RNA was extracted after 4, 8, and 12 h. *Error bars* represent the standard deviation of three biological replicates. **c** Expression levels of *sxlr* in RUT-C30, the *xyr1* deletion mutant (Δ*xyr1*), and the *xyr1*-overexpression strain (O*xyr1*); and *xyr1* expression levels in RUT-C30, Δ*sxlr*, and O*sxlr* when wheat bran and Avicel were used as the carbon source. Expression levels were normalized to the signal of β-actin. RNA was extracted after 4, 8, and 12 h. *Error bars* represent the standard deviation of three biological replicates

To further identify the regulation mechanism of SxlR on xylanase activity, we examined the expression levels of five xylanase-encoding genes using qPCR; the xylanases encoded by these genes included three members of GH11 family (XYN1, XYN2, and newly discovered XYN5), a GH10 family member XYN3 and a GH30 family member XYN4 [23]. The transcription levels of the genes encoding the three GH11 members showed significant differences in the Δ*sxlr* strain (upregulated) and the O*sxlr* strain (downregulated) at all sampling time points using wheat bran and Avicel were used as inducers (Fig. 5a).

The transcription level of the genes encoding XYN3 and XYN4 were also downregulated in the O*sxlr* strain at all sampling time points (Fig. 5a). However, their transcription levels in the Δ*sxlr* strain changed differently (Fig. 5a).

In the Δ*sxlr* strain, the relative expression level of *xyn3* was upregulated compared to the wild-type stain in the first 8 h of incubation in the inducing medium. The relative expression level of *xyn4* was upregulated after 4 h of induction, but downregulated after 8 and 12 h of induction (Fig. 5a). When using xylan as the inducer, the transcription level variation tendency of the encoding genes of XYN1, XYN2, XYN5, and XYN3 was similar to that using wheat bran and Avicel as the inducer (Fig. 5b). In contrast, the relative expression level of *xyn4* was upregulated in the O*sxlr* strain after 4 h of induction (Fig. 5b).

To further confirm the qRT-PCR results, qRT-PCR experiments were re-carried out using *rpl6e* (a ribosomal protein encoding gene) [24] and *sar1* (a small GTPase encoding gene) [25] as the reference gene. The transcription level variation tendency of the five xylanase-encoding genes was similar to that using β-actin gene as the reference gene (Additional file 3).

Additionally, we examined the relative expression levels of other genes coding (hemi-) cellulases or their regulators, including three major cellulase genes (*cbh1*, *cbh2*, *egl1)*, the key transcriptional activator *(xyr1)*, and two hemi-cellulase genes (β-mannanase, *man1* and α-l-arabinofuranosidase, *abf1*). There were no significant differences in the relative expression levels of these genes between RUT-C30 strain and Δ*sxlr* strain (Additional file 3). These results provide the first experimental evidence that SxlR is a negative and specific regulator of GH11 family xylanases.

Xyr1, a major transcription activator in *T. reesei*, regulates expression of most cellulase and hemi-cellulase genes directly, including xylanases [26, 27]. To investigate the relationship between Xyr1 and SxlR, we determined the relative expression levels of *sxlr* in the Δ*xyr1* strain (an *xyr1*-deletion strain derived from RUT-C30) and in the O*xyr1* strain (an *xyr1*-overexpression strain derived from RUT-C30) as well as the relative expression levels of *xyr1* in Δ*sxlr* strain and O*sxlr* strain (Fig. 5c). Comparing with *xyr1*, the transcription level of *sxlr* was quite low in RUT-C30. The *xyr1* transcription level decreased significantly in the O*sxlr* strain at all the sampling time points. In contrast, its transcription level in the Δ*sxlr* strain significantly increased at 4-h induction, and then decreased from 8-h induction. It seemed that SxlR might repress *xyr1* expression somehow. The transcription level of *sxlr* in the O*xyr1* strain was nearly the same as that in RUT-C30 strain, and its transcription level in the Δ*xyr1* strain was lower or similar to that in RUT-C30 strain. The variation tendency of the *sxlr* transcription level was quite different from that of the downstream genes of Xyr1, of which the transcription were sharply repressed after the deletion of Xyr1 [27].

### SxlR binds the promoters of GH11 xylanase genes

**Fig. 6:**
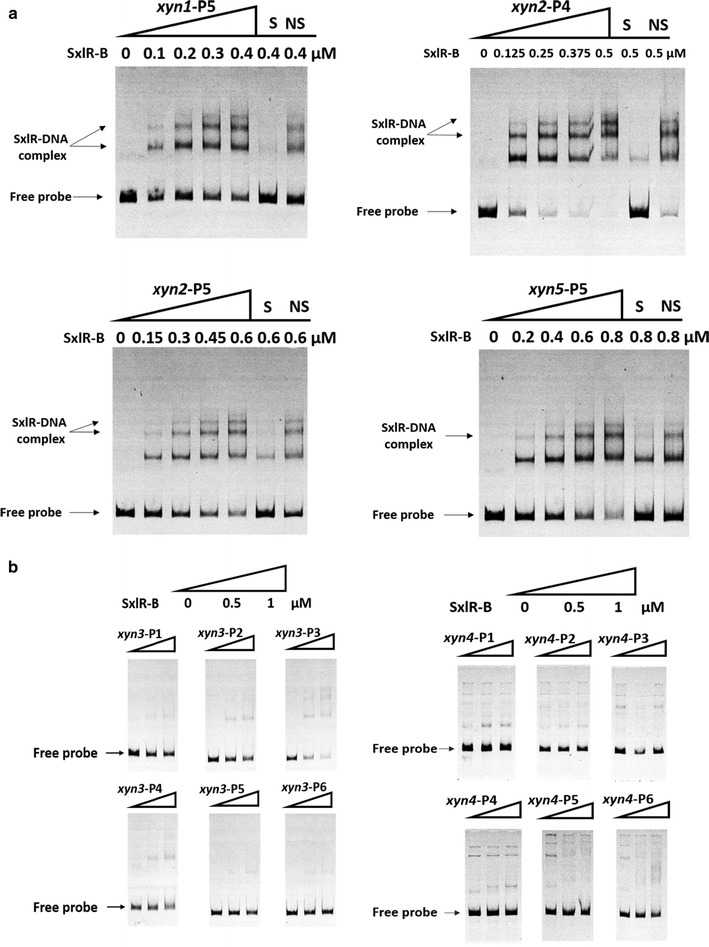
EMSAs of SxlR binding to the promoter regions of xylanase genes. **a** DNA binding of SxlR to the promoter regions of *xyn1*, *xyn2*, and *xyn5*. The amounts of purified SxlR binding domain (SxlR-B, μM) used were as indicated; ~10 ng of Cy5-labeled probe was added to each reaction. The shifts were verified to be specific by adding 100-fold excess of unlabeled specific (S) and non-specific (NS) competitor DNA. The SxlR-DNA complex is indicated by the *arrow*. **b** DNA binding of SxlR to the *xyn3* and *xyn4* promoter regions. We used three concentrations of SxlR-B: 0, 0.5, and 1 μM; ~10 ng of Cy5-labeled probe was added to each reaction

According to functional domain analysis, SxlR contains two distinct conserved domains, one GAL4-like Zn_2_Cys_6_ binuclear cluster DNA binding domain at the N-terminus (cd00067: residues 282–317) and one fungal transcription factor regulatory middle homology region at the C-terminus (cd12148: residues 436–853). To verify the regulation of the GH11 xylanase genes by SxlR, the GAL4-like Zn_2_Cys_6_ binuclear cluster DNA binding domain was expressed in vitro for EMSAs. The nearly 1500-bp upstream regions (nucleotide position −1500 to −1) assumed to be the promoter regions of the xylanase-encoding genes were divided into six parts (for example, *xyn2*-P1, 268 bp, nucleotide position −268 to −1; *xyn2*-P2, 268 bp, position −516 to −249; *xyn2*-P3, 268 bp, position −764 to −497; *xyn2*-P4, 268 bp, position –1012 to −745; *xyn2*-P5, 268 bp, position −1260 to −993; *xyn2*-P6, 268 bp, position −1508 to −1241). Based on the EMSA gel shifts, SxlR could bind the *xyn1*-P5, *xyn2*-P4, *xyn2*-P5, and *xyn5*-P5 promoter regions of the three GH11 xylanases (Fig. 6a). No specific gel shift was observed for the *xyn3* and *xyn4* promoters, which belonged to the GH10 and GH30 families, respectively (Fig. 5b; Additional file 4). It means that SxlR plays a critical role in the inhibition of GH11 family genes through binding to their promoter regions directly.

**Fig. 7:**
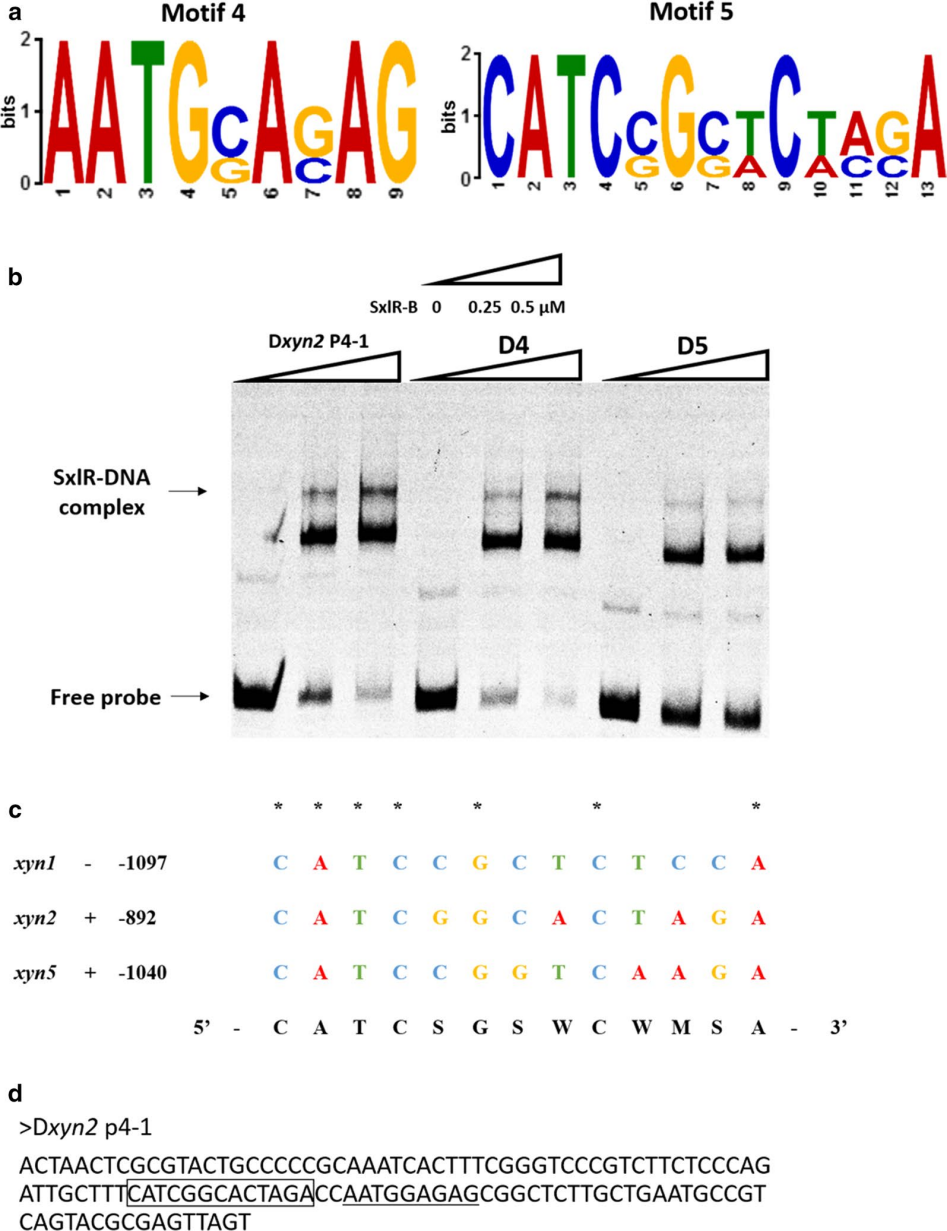
Identification of the SxlR binding consensus sequence. **a** Sequence motifs of a putative SxlR binding consensus sequence derived by MEME from D*xyn2*-P4-1, D*xyn1*-P5-2 and D*xyn*5-P5-2. Two putative SxlR binding consensus sequences were obtained. **b** EMSA results of SxlR binding to D*xyn2* P4-1, D4 (Motif 4 deletion), and D5 (Motif 5 deletion); the SxlR-DNA complex is indicated by the *arrow*. The amounts of purified SxlR binding domain (SxlR-B, μM) used were as indicated; ~10 ng of Cy5-labeled probe was added to each reaction. **c** Alignment of SxlR binding consensus sequence on sense (+) or antisense (−) strand in the upstream regions of *xyn1*, *xyn2*, and *xyn5*. The *numbers following the gene name* indicated the point of the 5′ starting nucleotide relative to the translation start point and the same nucleotide was indicated by *asterisk*. **d** The location of motif 4 and motif 5 in D*xyn2* P4-1 probe. Motif 4 was indicated by *underline* while motif 5 was indicated by *pane*

To identify the binding motif of SxlR, three promoter fragments (*xyn2*-P4, *xyn1*-P5, and *xyn5*-P5) were each divided into two 144-bp segments (Additional file 5A). SxlR bound to *xyn2*-P4-1, *xyn1*-P5-2, and *xyn5*-P5-2 (Additional file 5B). Based on MEME Suite (http://meme-suite.org/tools/meme) analysis, three candidate consensus motifs were predicted (Additional file 5C, D). However, the deletion of these three motifs did not affect the binding of SxlR to these DNA fragments (Additional file 5E). The 144 -bp fragments were further shortened for identification of a consensus motif, and two motifs were predicted (Fig. 7a). Based on the disappearance of the SxlR-DNA complex with the deletion of motif 5 (Fig. 7b), the consensus motif of SxlR was determined as 5′-CATCSGSWCWMSA-3′ (Fig. 7c, d). In this motif, one guanine and one cytosine are present in the core region and the consistent nucleotides are located in the flanks. The potential SxlR binding motif were not detected in the promoter regions of Xyr1 and xylanases XYN3 and XYN4. It suggested that SxlR might not regulate the transcription of Xyr1 and the GH10 family member XYN3 as well as the GH30 family member XYN4 directly.

## Discussion

The aim of this study was to gain a comprehensive understanding of how viable SAN progeny produces higher levels of xylanases [17]. We focused on candidate TFs exhibiting differential expression levels between SAN and euploid progeny (six TF-encoding genes: ID in jgi|Trire2: 106677, 65854, 111446, 68930, 111515, 36913), as well as another candidate TF, SxlR. We confirmed that SxlR is a negative regulator of GH11 family xylanases. However, overexpression of the other six tested TFs had no effect on the xylanase activity. It seems that the segmental aneuploidy did not result in a change in *sxlr* gene copy number; however, it is still unknown whether segmental aneuploidy affects the expression of *sxlr* in SAN progeny.

Recently, another negative regulator of hemi-cellulase, xylanase promoter-binding protein 1 (Xpp1) was reported in *T. reesei* [18]. Xpp1 regulates transcription of hemi-cellulase genes only at later stages of cultivation. There was no significant difference in xylanase activity between an *xpp1*-disrupted strain and its parent strain before 72 h [18]. In this study, SxlR regulated expression of GH11 xylanase genes during the entire induction period, and the xylanase activity of the deletion strain was significantly higher than that of the parent strain (Fig. 3a). It has been suggested that SxlR may play an important role in regulating xylanase expression in *T. reesei.* Xpp1 repressed the expression of *xyn1*, *xyn2*, and *bxl2* (encoding a putative β-xylosidase) but like SxlR, did not affect cellulase genes [18]. SxlR bound the *xyn1*, *xyn2*, and *xyn5* promoter regions in vitro (Fig. 6a) and was demonstrated to be a GH11-specific TF. This indicated that the regulation mechanisms of SxlR and Xpp1 are not identical. Additionally, in inducing medium containing xylan, the *xpp1*-disrupted strain showed nearly 2.2-fold increases in transcription levels of *xyn2* at 72 h, while the Δ*sxlr* strain showed 9.9-, 2.1-, and 4.6-fold increases in transcription levels of *xyn2* at 4, 8, and 12 h, respectively. Both the intensity and timing of these two TFs differed.

The binding consensus sequence of SxlR (5′-CATCSGSWCWMSA-3′) differs from that of the hemi-cellulase regulator Xpp1 (a hexameric palindrome 5′-WCTAGW-3′ together with an inverted AGAA-repeat [18]). In the *xyn1* promoter region, the binding consensus sequence of Xpp1 is located in the *xyn1*-P3 fragment, while SxlR binds to *xyn1*-P5. In the *xyn2* promoter region, the binding consensus sequence of Xpp1 is located in *xyn2*-P1 fragment, while SxlR binds to *xyn2*-P4 and *xyn2*-P5. In the *xyn5* promoter region, only the SxlR binding consensus sequence was found. Meanwhile, in the *bxl2* promoter region, only the Xpp1 binding consensus sequence was found. Therefore, SxlR has a regulon that differs from that of Xpp1.

Most xylanases (endo-β-1, 4-xylanase, EC 3. 2. 1. 8) belong to the GH10 and GH11 families. Compared to GH10, the GH11 family is much more xylan-specific [28]. In *T. reesei*, XYN2 belonging to GH11 family was the dominant extracellular xylanase. The Δ*sxlr* strain grew more vigorously than the wild-type on MM with xylan or xylose as the sole carbon source. This indicates that the deletion of *sxlr* results in the high ability to utilize xylan or xylose. According to our hypothesis, the role of SxlR is the downregulation of the main xylanases to save energy when cellulose or glucose is present in the habitat of *T. reesei*. Hexose is, after all, the preferred carbon resource of these organisms [29]. The novel TF SxlR is indicative of the sophisticated regulatory network evolved by *T. reesei* to adapt to complex environments.

Unlike the previously reported global regulators of carbohydrate-active enzymes (CAZymes), SxlR, as a specific regulator of GH11 family xylanases, might provide new leads for strain engineering to enhance hemi-cellulase activity in *T. reesei.* In the straw hydrolysis assay, more reducing sugar was produced by the Δ*sxlr* culture supernatant than by the parent strain. Compared with steam-exploded rice straw, higher straw hydrolysis was achieved by steam-exploded rice and corn straw mixture (Fig. 4d). This was probably because the hemi-cellulose content of corn straw was much higher than that of rice straw (41% vs 22%) [30, 31]. It has been suggested that the utilization of hemi-cellulose-rich substrate was significantly improved by the modified enzyme preparation. Because most TFs regulate cellulase and hemi-cellulase genes concurrently [32], the ratio of cellulase and hemi-cellulase is difficult to optimize by genetic manipulation of TFs. Our results shown that the appropriate proportion of the enzyme preparation can be conveniently optimized via engineering of *sxlr*. The derived strain would have a higher xylanase activity, while the cellulase activity would not be affected.

Through phylogenetic analysis of SxlR, we found its homologous genes in other cellulase producer including *Trichoderma virens* (XP_013961599.1), *T. atroviride* (XP_013948752.1), *Neurospora crassa* (XP_960943.2), *Penicillium oxalicum* (EPS34484.1), *Aspergillus nidulans* (XP_681446.1), *A. oryzae* (KOC12472.1), and *A. niger* (CAK40371.1), which indicates that its function may be conserved (Additional file 6).

## Conclusions

SxlR appears to be a major xylanase regulator in *T. reesei* that represses the expression of xylanases belonging to the GH11 family and does not affect cellulase activity. Deletion of *sxlr* dramatically increases xylanase activity. SxlR is a good target candidate for *T. reesei* strain modification, especially for hydrolyzing substrates rich in hemi-cellulose.

## Methods

### Strains and culture conditions

*Trichoderma reesei* strains including QM6a (ATCC 13631) and RUT-C30 (ATCC 56765) were maintained on potato dextrose agar plate (PDA) at 28 °C for 7 days for spore collection. *Escherichia coli* DH5α used as cloning host was culture at 37 °C in Luria–Bertani (LB) medium. *Agrobacterium tumefaciens* AGL1 was used to transform the gene to *T. reesei* strains. To induce enzyme production, the conidial suspension (0.5 mL, 1 × 10^7^ conidia/mL) was inoculated into a 50-mL Erlenmeyer flask containing 10 mL of Sabouraud Dextrose Broth (SDB) and incubated for 40 h on an orbital shaker at 200 rpm at 28 °C. The culture was then transferred into a flask containing 10 mL of inducing fermentation medium at 10% inoculum ratio (v/v). The flasks were incubated on an orbital shaker at 200 rpm at 28 °C for 1 week. The wheat bran and Avicel medium were prepared as follows: 0.4% KH_2_PO_4_, 0.28% (NH_4_)_2_SO_4_, 0.06% MgSO_4_·7H_2_O, 0.05% CaCl_2_, 0.06% urea, 0.3% tryptone, 0.1% Tween-80, 0.5% CaCO_3_, 0.001% FeSO_4_·7H_2_O, 0.00032% MnSO_4_·H_2_O, 0.00028% ZnSO_4_·7H_2_O, 0.0004% CoCl_2_, 2% wheat bran, 3% microcrystalline cellulose. We also used minimal medium (MM) containing 0.5% (NH_4_)_2_SO_4_, 1.5% KH_2_PO_4_, 0.06% MgSO_4_, 0.06% CaCl_2_, 0.0005% FeSO_4_·7H_2_O, 0.00016% MnSO_4_·H_2_O, 0.00014% ZnSO_4_·7H_2_O, and 0.0002% CoCl_2_ with 1% lactose or 1% xylan used as carbon source for 3 days of fermentation.

### Construction of Δ*sxlr* in *T. reesei* QM6a strain using the CRISPR/Cas9 system

*T. reesei* 6a-u (a uridine-dependent strain derived from 6a-pc) [19] was used as host. The guide RNA (gRNA) cassette including the synthetic gRNA sequence and target DNA of the *sxlr* gene (5′-GGGCAAGCCTCGCAAGCGGT*tgg*-3′, PAM is shown in italics) was driven by T7 promoter and transcribed into RNA in vitro with the MEGAscript T7 Kit (Ambion, Austin, TX, USA). Donor DNA-*sxlr* (dDNA-*sxlr*) containing the 5′- and 3′ flanking sequences of *sxlr* (jgi|Trire2|123881) and the selectable marker cassette (the *ura5* gene from *P. oxalicum* controlled by the Pgpda promoter and Ttrpc terminator, Pgpda-*poura5*-Ttrpc) was ligated into the pMD-18T vector (Takara, Dalian, China). The gRNA and dDNA-*sxlr* were co-transformed using a modified polyethylene glycol-mediated protoplast transformation procedure [33]. The transformants were selected using MM plates with 1% glucose as the carbon source.

### Deletion, overexpression, and re-complementation of *sxlr* in *T. reesei* RUT-C30

To delete the *sxlr* gene (jgi|TrireRUTC30_1|26638) of *T. reesei*, the 2.8-kb *sxlr* coding region was replaced by the *hph* (hygromycin phosphotransferase) gene. This was performed by amplifying 1.5 kb from upstream and 1.7 kb from downstream of *sxlr* from genomic DNA of *T. reesei* using the primer pairs listed in Additional file 7. Then, the two resulting PCR fragments were ligated into the *Hind*III (upstream) and *Xho*I (downstream) sites of the linearized pXBthg vector [30] using the ClonExpress II One Step Cloning Kit (Vazyme, Nanjing, China).

For overexpression of *sxlr* under a strong constitutive promoter in *T. reesei*, we fused the *T. reesei* translation-elongation factor 1α (*tef1)* promoter, the *sxlr* coding region and the Ttrpc terminator from *A. nidulans* and inserted this fragment into the *Hind*III and *Xba*I sites of pXBthg using the ClonExpress II One Step Cloning Kit. Besides, we overexpressed 76601, 83920, 89588, 133726, 26508, and 43491 (gene ID in jgi|TrireRUTC30_1) with the same method.

For re-complementation of *sxlr in* the Δ*sxlr* strain, the promoter, coding region, and terminator of *sxlr* were inserted into the *Hind*III and *Xba*I sites of the pXBt vector [the sequence is similar to pXBthg, except the *hph* marker gene was replaced by the bleomycin resistance (*ble*) gene] and the sequence downstream of *sxlr* was inserted into the *Xho*I site by the same method mentioned above.

All vectors constructed were verified by sequencing. *Agrobacterium*-mediated transformation was conducted as described previously by Ma et al. [34].

### Biochemical assays

FPAase activities were determined using cellulose filter paper as described previously [35]. Xylanase activities were tested using 2% xylan from beechwood (Sigma, St. Louis, USA) as described previously [36]. Protein concentrations in the culture supernatant were determined by RC-DC protein assay (Bio-Rad, Hercules, CA, USA) (1976). Biomass was tested by the diphenylamine colorimetric method [37].

For straw hydrolysis, different pretreated biomasses such as rice straw and a corn stover plus rice straw mixture (3:7 ratio) [38] were used as substrates. The standard hydrolysis assay was carried out in a volume of 1 mL of 50 mM sodium acetate, pH 4.8, and 5% (w/v) pretreated straw as substrate in a 2-mL FastPrep tube (MP Biomedicals, Santa Ana, CA, USA). The enzyme dosage was 20 U of cellulase activity/g substrate. Incubation was typically at 50 °C with shaking at 200 rpm for 3 days, followed by centrifugation at 12,000 rpm for 10 min to obtain the supernatant to determine the reducing sugar with DNS. Each sample was examined in triplicate.

### Growth of *sxlr* transformants on plates

Spores of *T. reesei* RUT-C30, Δ*sxlr,* O*sxlr*, and R*sxlr* were first prepared by growth on PDA plates at 28 °C and harvesting spores after 7 days. Spores were counted using a hemocytometer and 10^5^ spores of each strain were inoculated onto PDA plates and MM plates containing 1% xylose, xylan, glucose, lactose, or Avicel. Double-layer Avicel plates were prepared by first casting an MM agarose bottom layer containing no carbon source, followed by an MM agarose top layer containing 1% Avicel; each strain was grown in triplicates. These plates were incubated at 28 °C before phenotypic examination.

### Protein identification with MALDI-TOF/TOF analysis

After 7 days’ culture with wheat bran and Avicel medium, the supernatant of RUT-C30, Δ*sxlr*, O*sxlr*, and R*sxlr* strain was collected. The samples were loaded into 12.5% PAGE gel, 70 min at 120 V were preferred for proteins separation. Coomassie brilliant blue R250 (Sangon, Shanghai, China) was used to color the gel for 1 h. Destainer (distilled water: ethanol: acetic acid, 5:4:1, v/v) was added for another 2–4 h. Finally, the protein band was excised from the gel and send to Shanghai Applied Protein Technology Co. Ltd, protein analysis was conducted with a MALDI-TOF/TOF mass spectrometer 5800 Proteomics Analyzer (Applied Biosystems, Framingham, MA, USA), NCBI *Trichoderma* (59929 protein sequence) was selected as database.

### RNA extraction and real-time PCR

Mycelia were harvested after induction by wheat bran and Avicel for 4, 8, and 12 h, then transferred into TRIzol reagent (Invitrogen, USA) with standard protocol. Three biological replicates were prepared in the process. Real-time PCR was performed with primers listed in Additional file 7 with standard method using β-actin gene, *rpl6e* (a ribosomal protein encoding gene) and *sar1* (a gene encoding a small GTPase) as the reference genes, respectively.

### Expression and purification of the DNA binding domain of SxlR

The DNA binding domain (residues 252–347) of SxlR (SxlR-B) was expressed by the pGEX system according to the manufacturer’s guidelines. First-strand cDNA was used as a template to amplify the fragment encompassing the DNA binding domain of SxlR using the primers indicated in Additional file 7. The fragment was then ligated into plasmid pGEX-4T-1 via *Bam*HI and *Xho*I double digestion to produce pGEX-4T-SxlR-B, then subsequently introduced into *E. coli* BL21 (DE3) for protein production. The recombinant protein was purified using a GST-Bind resin column (Merck, Darmstadt, Germany) according to the supplier’s recommendations. SDS-PAGE was used to verify the purified protein.

### Electrophoretic mobility shift assays

For EMSA, the labeling of probes containing the promoter regions of specific genes was performed via PCR using the primers listed in Additional file 7. The nearly 1500-bp upstream regions (nucleotide position −1500 to −1) assumed to be the promoter regions of the xylanase-encoding genes from *T. reesei* RUT-C30 were divided into six parts and each part was about 268 bp (Additional file 4A). The genomic DNA of *T. reesei* RUT-C30 was used as the template and the PCR products were purified by gel electrophoresis and quantified using a BioPhotometer plus (Eppendorf, Hamburg, Germany). The experiment was then performed as described previously by Chen et al. [39]. Motif 1 (5′-AMTGSAGAG-3′) was located in −1034 to −1026 position of *xyn1* promoter, −890 to −882 position of *xyn2* promoter and −1091 to −1083 position of *xyn5* promoter. Motif 2 (5′-TGAWGAG-3′) was located in −1022 to −1016 position of *xyn1* promoter, −957 to −951 position of *xyn2* promoter and −1021 to −1015 position of *xyn5* promoter. Motif 3 (5′-WTATAT-3′) was located in −998 to −993 position of *xyn2* promoter and −1117 to −1112 position of *xyn5* promoter. Motif 4 (5′-AATGSASAG-3′) was located in −1119 to −1111 position of *xyn1* promoter, −890 to −882 position of *xyn2* promoter and −1091 to −1083 position of *xyn5* promoter. Motif 5 (5′-CATCSGSWCWMSA-3′) was located in −1110 to −1098 position of *xyn1* promoter, −905 to −893 position of *xyn2* promoter and −1053 to −1041 position of *xyn5* promoter.

## Additional files

**Additional file 1.** Putative specific xylanase transcription factors in *T. reesei.*

**Additional file 2.** *sxlr* transformants verification. (A) PCR result for R*sxlr* transformants identification. Three different transformants were chosen to verify. (B) Verification of random insertion in R*sxlr* transformants. During the *Agrobacterium*-mediated transformation, the T-DNA will randomly insert into genome with LB and RB, so we can verify it with PCR. The expected PCR product length of 5′ and 3′ verification was 6.1 kb and 2.6 kb. The *sxlr* re-complementation vector was used as positive control. Three different transformants were chosen to verify. The *sxlr* transcription levels in *sxlr* transformants were normalized to the signal of β-actin (C) or rpl6e (D), a gene encoding a ribosomal protein. RNA was extracted after 12 h induction by wheat bran and Avicel. Error bars represent the standard deviation of three biological replicates.

**Additional file 3.** Quantitative PCR analysis of cellulase and hemi-cellulase gene expression levels. The expression level of five xylanase genes in RUT-C30, Δ*sxlr*, O*sxlr* and R*sxlr* when wheat bran and Avicel were used as carbon sources. Expression levels were normalized to the signal of rpl6e, (A) or sar1 (B), a gene encoding a small GTPase. (C) The expression levels of cbh1, cbh2, egl1, xyr1, man1 and abf1 in RUT-C30 and Δ*sxlr*. Expression levels were normalized to the signal of β-actin. RNA was extracted after 24, 48, and 72 h after induction by wheat bran and Avicel. Error bars represent the standard deviation of three biological replicates.

**Additional file 4.** The description of xylanase genes promoter and EMSAs of SxlR binding to *xyn3* and *xyn4*. (A) The nearly 1500-bp upstream regions (nucleotide position –1500 to –1) assumed to be the promoter regions of the xylanase-encoding genes from *T. reesei* RUT-C30 were divided into six parts and each part was about 268 bp. (B) DNA binding of SxlR to the *xyn3* and *xyn4* promoter regions. We used three concentrations of SxlR-B: 0, 0.5, and 1 μM; ~ 10 ng of Cy5-labeled probe was added to each reaction. For specific (S) and non-specific (NS) control experiment, 100-fold excess of unlabeled S and NS competitor DNA were added.

**Additional file 5.** Truncation prmoter sequence of GH11 xylanase genes. (A) The description of DNA sequence truncation. Each sequence was divided into two parts. (B) EMSAs of SxlR binding to *xyn2*-P4, *xyn1*-P5 and *xyn5*-P5 truncation sequence, respectively. (C) Three putative SxlR binding consensus sequences derived by MEME. (D)The location of three putative SxlR binding consensus sequences in *xyn2*-P4-1, *xyn1*-P5-2 and *xyn5*-P5-2. Motif 1, 2 and 3 was labeled as red, blue and green, respectively. D*xyn2*-P4-1, D*xyn1*-P5-2 and D*xyn5*-P5-2 was the sequence after truncation. (E) EMSAs of SxlR binding to D*xyn2*-P4-1, D*xyn1*-P5-2 and D*xyn5*-P5-2, the SxlR-DNA complex was indicated by *arrow*. The amounts of purified SxlR binding domain (SxlR-B, μM) used were as indicated; ~ 10 ng of Cy5-labeled probe was added to each reaction.

**Additional file 6.** Phylogenetic relationship between SxlR and putative orthologs. The phylogenetic tree was inferred using the neighbor-joining method. Evolutionary analyses were conducted in MEGA 5. The gene ID in NCBI is ETR97987.1 (*T. reesei*), XP_013961599.1 (*T. virens*), XP_013948752.1 (*T. atroviride*), XP_960943.2 (*Neurospora crassa*), EPS34484.1 (*Penicillium oxalicum*), XP_681446.1 (*Aspergillus nidulans*), CAK40371.1 (*A. niger*) and KOC12472.1 (*A. oryzae*).

**Additional file 7.** Oligonucleotides used in this study.

## Abbreviations

SxlR: specialized xylanase regulator
EMSA: electrophoretic mobility shift assay
TFs: transcriptional factors
CCR: carbon catabolite repression
SAN: segmentally aneuploid
Xpp1: xylanase promoter‑binding protein 1
CRISPR: clustered regularly interspaced short palindromic repeats
SDS-PAGE: sodium dodecyl sulfate–polyacrylamide gel electrophoresis
GH11: glycoside hydrolase family 11
CAZymes: carbohydrate-active enzymes
