# Supplementary material for: A novel transcription factor specifically regulates GH11 xylanase genes in Trichoderma reesei
Source: Biotechnol Biofuels. 2017 Aug 3;10:194. doi: 10.1186/s13068-017-0878-x (PMC5541735; doi:10.1186/s13068-017-0878-x)
Supplement: Supplementary file 1 — Additional file 1. Putative specific xylanase transcription factors in T. reesei. [file 13068_2017_878_MOESM1_ESM.docx]

| **Gene ID in**  **RUT-C30** | **Gene ID in**  **QM6a** | **Chromosome**  **QM6a** | **Number on chromosome** | **Original scaffold**  **QM6a** | **Scaffold position**  **QM6a** | **Annotation** |
| --- | --- | --- | --- | --- | --- | --- |
| **76601** | **106677** | **2** | **35** | **7** | **1311494-1313703** | **Zn_2_Cys_6_ transcriptional regulator** |
| **83920** | **65854** | **4** | **948** | **17** | **50427-** **52658** | **Zn_2_Cys_6_ transcriptional regulator** |
| **89588** | **111446** | **3** | **1673** | **27** | **41968-45217** | **Zn_2_Cys_6_ transcriptional regulator** |
| **133726** | **68930** | **3** | **1619** | **27** | **193632-195569** | **Zn_2_Cys_6_ transcriptional regulator** |
| **26508** | **111515** | **3** | **1615** | **27** | **207908-209899** | **Zn_2_Cys_6_ transcriptional regulator** |
| **43491** | **36913** | **1** | **1339** | **69** | **6230-7984** | **Zn_2_Cys_6_ transcriptional regulator** |
| ***26638(sxlr)*** | **123881(*sxlr*)** | **5** | **686** | **28** | **76992-79757** | **Zn_2_Cys_6_ transcriptional regulator** |

**Additional file 1**

**The putative specific xylanase transcription factors in *Trichoderma reesei***
