# Supplementary figures and images for: A novel transcription factor specifically regulates GH11 xylanase genes in Trichoderma reesei
Source: Biotechnol Biofuels. 2017 Aug 3;10:194. doi: 10.1186/s13068-017-0878-x (PMC5541735; doi:10.1186/s13068-017-0878-x)

**Additional file 2
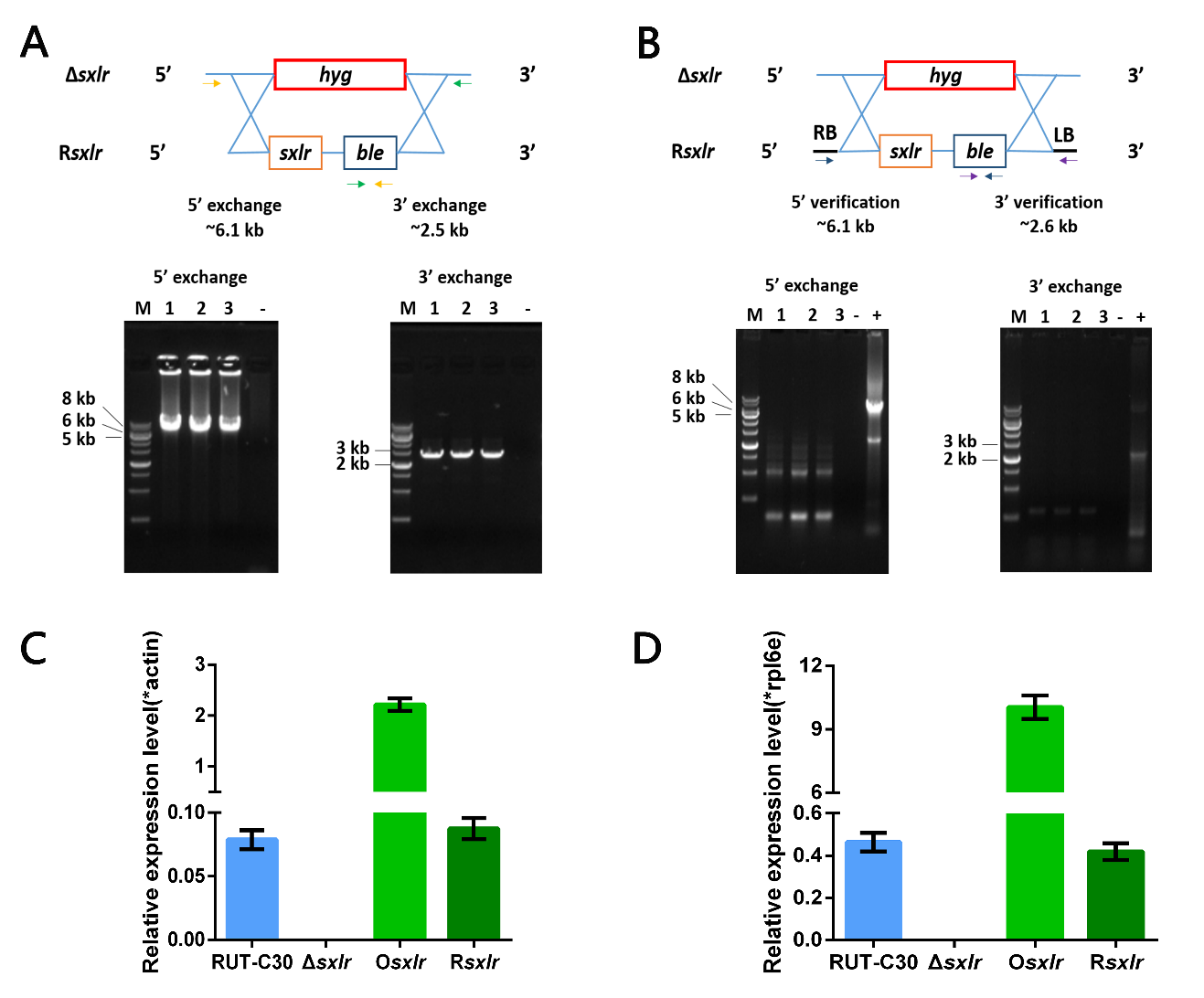
**

Supplement: Supplementary file 2 — Additional file 2. sxlr transformants verification. (A) PCR result for Rsxlr transformants identification. Three different transformants were chosen to verify. (B) Verification of random insertion in Rsxlr transformants. During the Agrobacterium-mediated transformation, the T-DNA will randomly insert into genome with LB and RB, so we can verify it with PCR. The expected PCR product length of 5′ and 3′ verification was 6.1 kb and 2.6 kb. The sxlr re-complementation vector was used as positive control. Three different transformants were chosen to verify. The sxlr transcription levels in sxlr transformants were normalized to the signal of β-actin (C) or rpl6e (D), a gene encoding a ribosomal protein. RNA was extracted after 12 h induction by wheat bran and Avicel. Error bars represent the standard deviation of three biological replicates. [file 13068_2017_878_MOESM2_ESM.docx]

**Additional file 3**


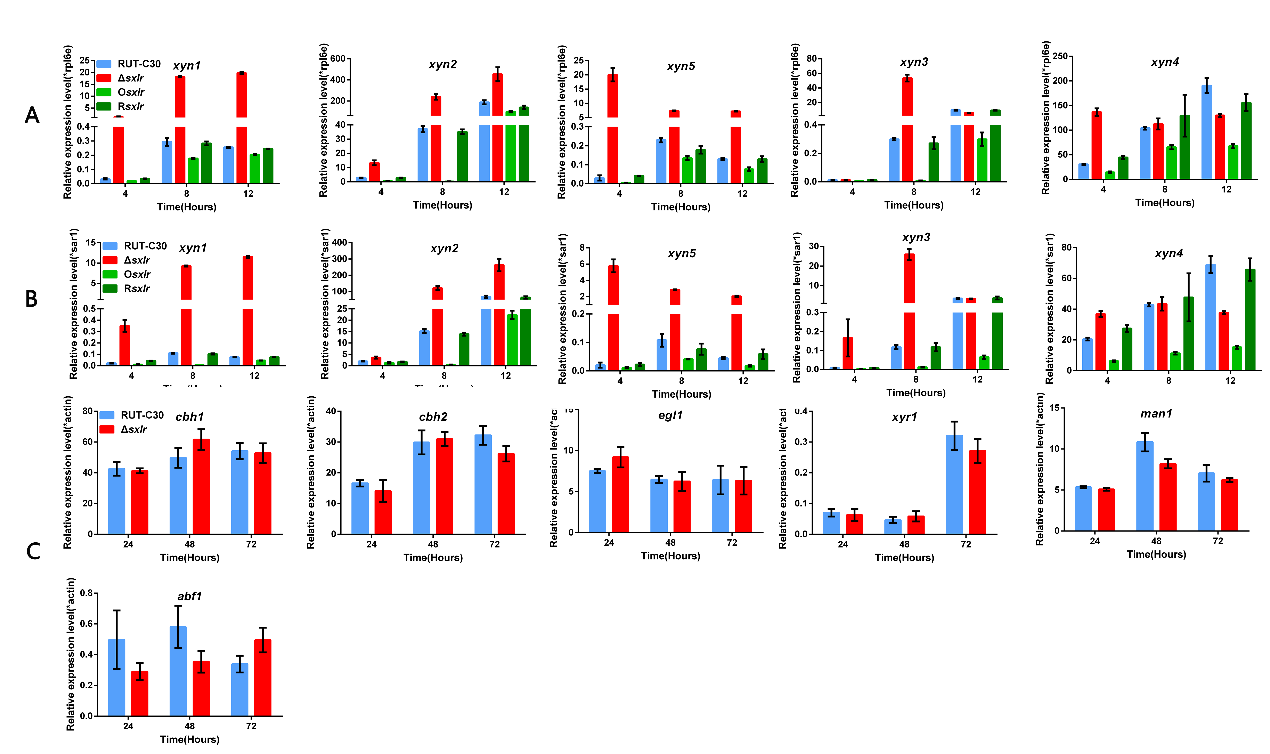

Supplement: Supplementary file 3 — Additional file 3. Quantitative PCR analysis of cellulase and hemi-cellulase gene expression levels. The expression level of five xylanase genes in RUT-C30, Δsxlr, Osxlr and Rsxlr when wheat bran and Avicel were used as carbon sources. Expression levels were normalized to the signal of rpl6e, (A) or sar1 (B), a gene encoding a small GTPase. (C) The expression levels of cbh1, cbh2, egl1, xyr1, man1 and abf1 in RUT-C30 and Δsxlr. Expression levels were normalized to the signal of β-actin. RNA was extracted after 24, 48, and 72 h after induction by wheat bran and Avicel. Error bars represent the standard deviation of three biological replicates. [file 13068_2017_878_MOESM3_ESM.docx]

**Additional file 4**

**
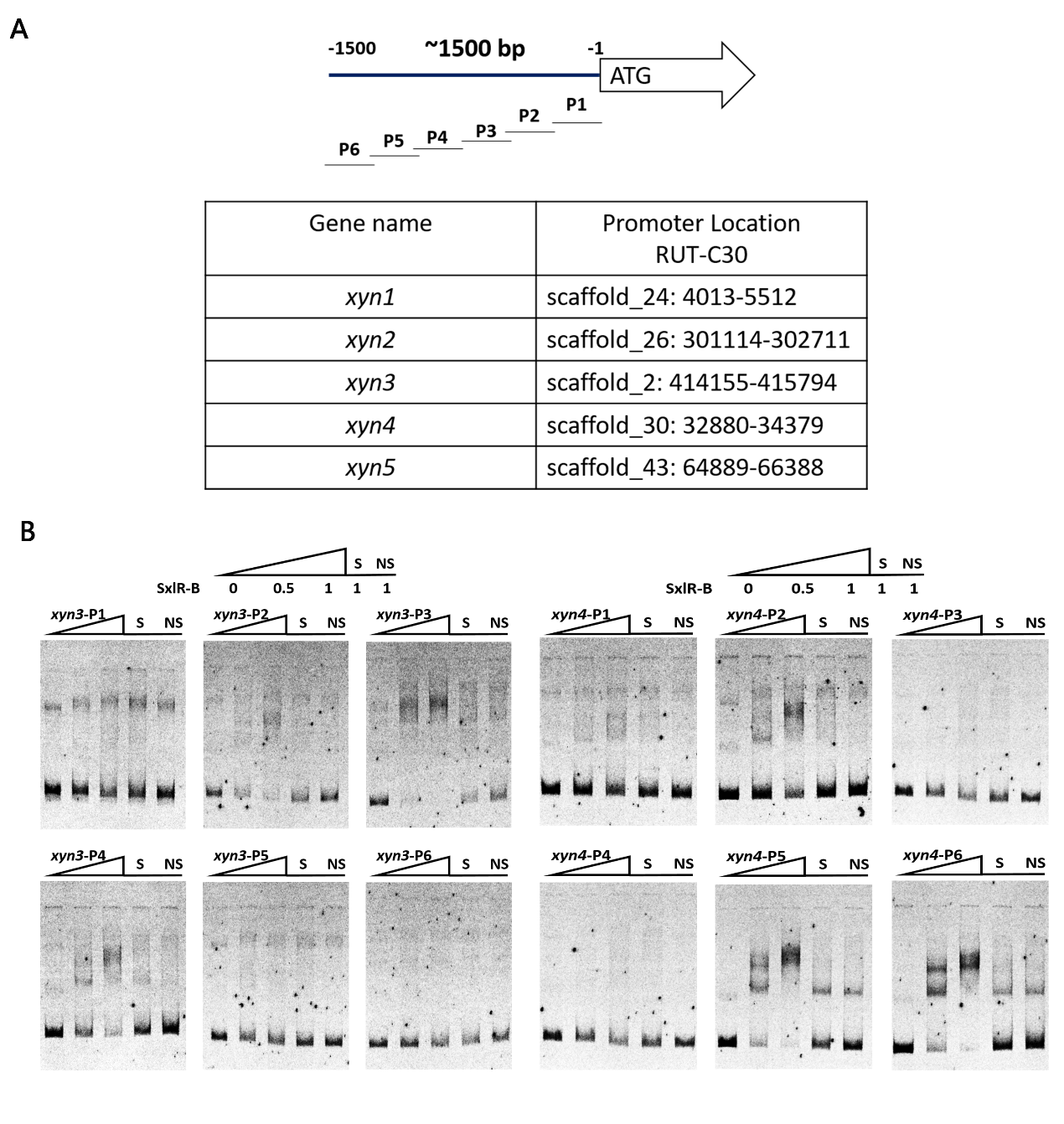
**

Supplement: Supplementary file 4 — Additional file 4. The description of xylanase genes promoter and EMSAs of SxlR binding to xyn3 and xyn4. (A) The nearly 1500-bp upstream regions (nucleotide position –1500 to –1) assumed to be the promoter regions of the xylanase-encoding genes from T. reesei RUT-C30 were divided into six parts and each part was about 268 bp. (B) DNA binding of SxlR to the xyn3 and xyn4 promoter regions. We used three concentrations of SxlR-B: 0, 0.5, and 1 μM; ~ 10 ng of Cy5-labeled probe was added to each reaction. For specific (S) and non-specific (NS) control experiment, 100-fold excess of unlabeled S and NS competitor DNA were added. [file 13068_2017_878_MOESM4_ESM.docx]

**Additional file 5**


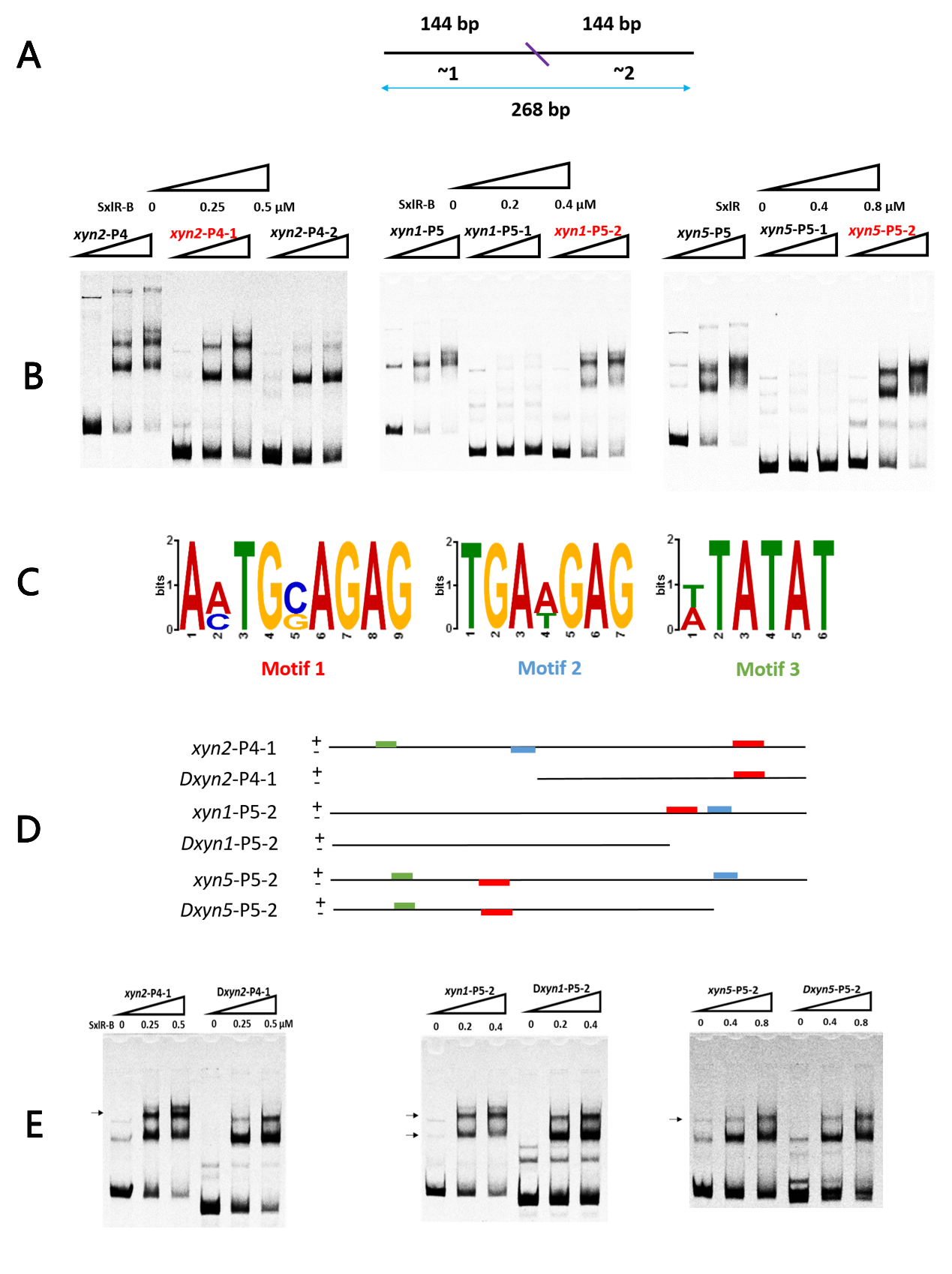

Supplement: Supplementary file 5 — Additional file 5. Truncation prmoter sequence of GH11 xylanase genes. (A) The description of DNA sequence truncation. Each sequence was divided into two parts. (B) EMSAs of SxlR binding to xyn2-P4, xyn1-P5 and xyn5-P5 truncation sequence, respectively. (C) Three putative SxlR binding consensus sequences derived by MEME. (D)The location of three putative SxlR binding consensus sequences in xyn2-P4-1, xyn1-P5-2 and xyn5-P5-2. Motif 1, 2 and 3 was labeled as red, blue and green, respectively. Dxyn2-P4-1, Dxyn1-P5-2 and Dxyn5-P5-2 was the sequence after truncation. (E) EMSAs of SxlR binding to Dxyn2-P4-1, Dxyn1-P5-2 and Dxyn5-P5-2, the SxlR-DNA complex was indicated by arrow. The amounts of purified SxlR binding domain (SxlR-B, μM) used were as indicated; ~ 10 ng of Cy5-labeled probe was added to each reaction. [file 13068_2017_878_MOESM5_ESM.docx]

**Additional file 6**


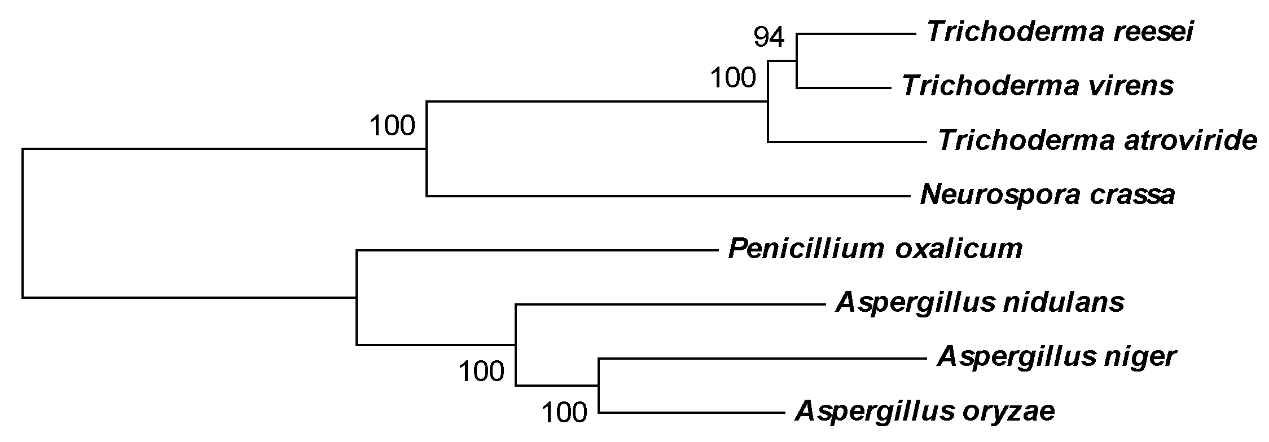

Supplement: Supplementary file 6 — Additional file 6. Phylogenetic relationship between SxlR and putative orthologs. The phylogenetic tree was inferred using the neighbor-joining method. Evolutionary analyses were conducted in MEGA 5. The gene ID in NCBI is ETR97987.1 (T. reesei), XP_013961599.1 (T. virens), XP_013948752.1 (T. atroviride), XP_960943.2 (Neurospora crassa), EPS34484.1 (Penicillium oxalicum), XP_681446.1 (Aspergillus nidulans), CAK40371.1 (A. niger) and KOC12472.1 (A. oryzae). [file 13068_2017_878_MOESM6_ESM.docx]
