# Supplementary material for: A novel transcription factor specifically regulates GH11 xylanase genes in Trichoderma reesei
Source: Biotechnol Biofuels. 2017 Aug 3;10:194. doi: 10.1186/s13068-017-0878-x (PMC5541735; doi:10.1186/s13068-017-0878-x)
Supplement: Supplementary file 7 — Additional file 7. Oligonucleotides used in this study. [file 13068_2017_878_MOESM7_ESM.docx]

**Additional file 7**

| **Name** | **Sequence(5’ to 3’)** |
| --- | --- |
| Usxlr F | ACGACGGCCAGTGCCAAGCTTCGGCGAACAAGCAGCCTACC |
| Usxlr R | GACCTGCAGGCATGCAAGCTTGCAGCAAGAAGAACCGAACC |
| Dsxlr F | CCGTCACCAGCCCTGCTCGAGCGTTTAGATCGCTGCCCACT |
| Dsxlr R | ATTATTATGGAGAAACTCGAGGTCACCTTGGCGACCACCTT |
| Ptef1 F | ACGACGGCCAGTGCCAAGCTTGGACAGAATGTACAGTACTATAC |
| Ptef1 R | GACGGTTTGTGTGATGTAGC |
| sxlr F | GCTACATCACACAAACCGTCATGTCACGTTATGGCAAGGGC |
| sxlr R | CTAGTCGCATCCGACATAGAC |
| Ttrpc F | TCTATGTCGGATGCGACTAGAGTAGATGCCGACCGGATCG |
| Ttrpc R | TAATTGCGCGGATCCTCTAGACAGGGCTGGTGACGGAATTTTC |
| RUsxlr F | ACGACGGCCAGTGCCAAGCTTGCTGGCATACTACTACTATG |
| RUsxlr R | GACCTGCAGGCATGCAAGCTTATCACGTGATCGATCGCGCG |
| RDsxlr F | CCGTCACCAGCCCTGCTCGAGCGTTTAGATCGCTGCCCACT |
| RDsxlr R | ATTATTATGGAGAAACTCGAGTGCCGTCCCTCGTCACGTCC |
| RT-xyn1 F | AAACTACCAAACTGGCGG |
| RT-xyn1 R | TTGATGGGAGCAGAAGATCC |
| RT-xyn2 F | CGGCTACTTCTACTCGTACTG |
| RT-xyn2 R | TTGATGACCTTGTTCTTGGTG |
| RT-xyn3 F | TACAAGGGCAAGATTCGTG |
| RT-xyn3 R | ACTGGCTTCCAATACCGT |
| RT-xyn4 F | GAACACCAACGGCGACAAC |
| RT-xyn4 R | CGGGCAAAGCGGAAGTATTG |
| RT-xyn5 F | CTACGGCTGGAGCACTAACC |
| RT-xyn5 R | CTGGTGACGCTGCCCTTC |
| EMSA-sxlr F | CGGGATCCTACGACGATGGAAGCCATTG |
| EMSA-sxlr R | CCGCTCGAGTCATAGATCAGCTGCTTCGAG |
| Cy5 xyn2 P4 F | ACTAACTCGCGTACTGGCCCCAGGGTCCGATTATAT |
| Cy5 xyn2 P4 R | ACTAACTCGCGTACTGAAGTAGAGTCAACCCGTATG |
| Cy5 xyn2 P4-1 F | ACTAACTCGCGTACTGGCCCCAGGGTCCGATTATAT |
| Cy5 xyn2 P4-1 R | ACTAACTCGCGTACTGTCAGCAAGAGCCGCTCTCCA |
| Cy5 xyn2 P4-2 F | ACTAACTCGCGTACTGTGGAGAGCGGCTCTTGCTGA |
| Cy5 xyn2 P4-2 R | ACTAACTCGCGTACTGAAGTAGAGTCAACCCGTATG |
| Cy5 xyn1 P5 F | ACTAACTCGCGTACTGACTCCATTGTCAACTTCACG |
| Cy5 xyn1 P5 R | ACTAACTCGCGTACTGAAGACTTGCCGGTGTTTAGA |
| Cy5 xyn1 P5-1 F | ACTAACTCGCGTACTGACTCCATTGTCAACTTCACG |
| Cy5 xyn1 P5-1 R | ACTAACTCGCGTACTGCAGTGAGCTCCCTTTGTCCG |
| Cy5 xyn1 P5-2 F | ACTAACTCGCGTACTGCGGACAAAGGGAGCTCACTG |
| Cy5 xyn1 P5-2 R | ACTAACTCGCGTACTGAAGACTTGCCGGTGTTTAGA |
| Cy5 xyn5 P5 F | ACTAACTCGCGTACTGCAATTCACATGGGATCTTTTAG |
| Cy5 xyn5 P5 R | ACTAACTCGCGTACTGTTGAGTCTATACCAAGTCCA |
| Cy5 xyn5 P5-1 F | ACTAACTCGCGTACTGCAATTCACATGGGATCTTTTAG |
| Cy5 xyn5 P5-1 R | ACTAACTCGCGTACTGTAGCATGTTGCAAAGGTTGA |
| Cy5 xyn5 P5-2 F | ACTAACTCGCGTACTGTCAACCTTTGCAACATGCTA |
| Cy5 xyn5 P5-2 R | ACTAACTCGCGTACTGTTGAGTCTATACCAAGTCCA |
| Cy5 Dxyn2 P4-1 F | ACTAACTCGCGTACTGCCCCCGCAAATCACTTTCGG |
| Cy5 Dxyn2 P4-2 F | ACTAACTCGCGTACTGTCAGCAAGAGCCGCTCTCCA |
| Cy5 Dxyn1 P5-2 F | ACTAACTCGCGTACTGCGGACAAAGGGAGCTCACTG |
| Cy5 Dxyn1 P5-2 R | ACTAACTCGCGTACTGATTCTTCTCTTGCCATTGGC |
| Cy5 Dxyn5 P5-2 F | ACTAACTCGCGTACTGTCAACCTTTGCAACATGCTA |
| Cy5 Dxyn5 P5-2 R | ACTAACTCGCGTACTGTCATTGACGGACTAGGGCATC |
| Ncy5 Dxyn2 P4-1 F | ACTAACTCGCGTACTGCCCCCGCAAATCACTTTCGG |
| Ncy5 Dxyn2 P4-1 R | ACTAACTCGCGTACTGACGGCATTCAGCAAGAGCCG |
